# Attitudes to and experiences with body weight control and changes in body weight in relation to all-cause mortality in the general population

**DOI:** 10.1371/journal.pone.0220838

**Published:** 2019-08-15

**Authors:** Camilla S. Morgen, Lars Ängquist, Merete Appleyard, Peter Schnohr, Gorm B. Jensen, Thorkild I. A. Sørensen

**Affiliations:** 1 National Institute of Public Health, University of Southern Denmark, Copenhagen, Denmark; 2 Department of Public Health, Section of Epidemiology, Faculty of Health and Medical Sciences, University of Copenhagen, Copenhagen, Denmark; 3 Novo Nordisk Foundation Centre for Basic Metabolic Research, Section on Metabolic Genetics, University of Copenhagen, Copenhagen, Denmark; 4 The Copenhagen City Heart Study, Bispebjerg and Frederiksberg Hospitals, The Capital Region, Copenhagen, Denmark; CUNY School of Public Health, UNITED STATES

## Abstract

**Background and aims:**

Increased body mass index (BMI = weight/height^2^; kg/m^2^) and weight gain is associated with increased mortality, wherefore weight loss and avoided weight gain should be followed by lower mortality. This is achieved in clinical settings, but in the general population weight loss appears associated with increased mortality, possibly related to the struggles with body weight control (BWC). We investigated whether attitudes to and experiences with BWC in combination with recent changes in body weight influenced long-term mortality among normal weight and overweight individuals.

**Population and methods:**

The study population included 6,740 individuals attending the 3^rd^ cycle in 1991–94 of the Copenhagen City Heart Study, providing information on BMI, educational level, health behaviours, well-being, weight half-a-year earlier, and answers to four BWC questions about caring for body weight, assumed benefit of weight loss, current and past slimming experiences. Participants reporting previous unintended weight loss (> 4 kg during one year) were excluded. Cox regression models estimated the associations of prior changes in BMI and responses to the BWC questions with approximately 22 years all-cause mortality with age as ‘time scale’. Participants with normal weight (BMI < 25.0 kg/m^2^) and overweight (BMI ≥ 25.0 kg/m^2^) were analysed separately, and stratified by gender and educational level, health behaviours and well-being as co-variables.

**Results:**

Compared with stable weight, weight loss was associated with significantly increased mortality in the normal weight group, but not in the overweight group, and weight gain was not significantly associated with mortality in either group. Participants with normal weight who claimed that it would be good for their health to lose weight or that they were currently trying to lose weight had significantly higher mortality than those denying it. There were no other significant associations with the responses to the BWC questions in either the normal weight or the overweight group. When combining the responses to the BWC questions with the weight changes, using the weight change as either a continuous or categorical variable, there were no significant interaction in their relation to mortality in either the normal weight or the overweight group.

**Conclusion:**

Attitudes to and experiences with BWC did not notably modify the association of changes in body weight with mortality in either people with normal weight or people with overweight.

## Introduction

Increased body mass index (BMI = weight/height^2^; kg/m^2^) as well as body weight gain is associated with increased mortality [1–4], so weight loss among people with overweight and avoidance of further weight gain is expected to be associated with lower mortality. Weight loss treatments in obese patients both by dietary interventions and bariatric surgery appears beneficial, also in terms of reduction in the associated excess mortality [5, 6]. However, the effects of weight loss in the general, more or less overweight adult population are often associated with increased mortality irrespective of whether it is declared intentional or not [3, 4, 7–11]. Keeping a stable body weight consistently appears to be associated with the lowest mortality.

Although underlying undiagnosed diseases or hazardous behaviours both inducing weight loss and enhancing mortality may confound the association, studies have so far not found indications of that kind of otherwise obvious risk of confounding [11, 12]. Whereas such unidentified confounding cannot be precluded, the findings leaves the option that weight loss may have a health damaging component mixed with the benefits, which makes the result a net balance between these effects. The studies have so far not identified increased risk of particular diseases as causes of the increased mortality, but it appears that the accompanying reduction of the lean body mass drives the association [13, 14]. This may outweigh the possible, but limited benefits from reducing the fat mass in people without obesity and reduce the benefits of weight loss in people with obesity, also after planned treatment aiming at weight loss [15].

Body weight gain and regain after weight loss is associated with stress [16–19], and may be implicated in risk of cardio-metabolic diseases such as diabetes, coronary heart disease and stroke [20]. Being preoccupied and struggling with and often failing the control of own body weight could perhaps be a stress factor [16, 21–23], which may be behind or worsen the associations between body weight changes and mortality. This raises the question whether the individual attitudes to and experiences with body weight control (BWC) alters mortality irrespective of any effects on health behaviours and actually achieved BWC. Our study aimed at answering the question separately for normal weight and for overweight people in the general population while considering their body weight changes, assuming that their attitudes to and experiences with BWC may depend on these aspects. We estimated all-cause mortality in a historical general population-based cohort in relation to participants’ responses to questions probing their attitudes to and experiences with BWC, while controlling for BMI, past and recent body weight changes and their educational level, health behaviours (smoking, alcohol drinking and physical activity) and well-being.

## Population and methods

The study population is defined as the Copenhagen City Heart Study [24], a cohort based on a random population sample of men and women aged 20 years and older, living in a part of the central municipality of Copenhagen (Østerbro) and first examined in 1976–78 (n = 14,223). This was followed by repeated health examinations including measurements of body height and weight (and hence BMI), and questions about educational level, smoking, alcohol intake, and physical activity. At each cycle of examination, the study invited additional individuals at age 20 and above to keep the broad coverage of adult age in the study.

**Fig 1:**
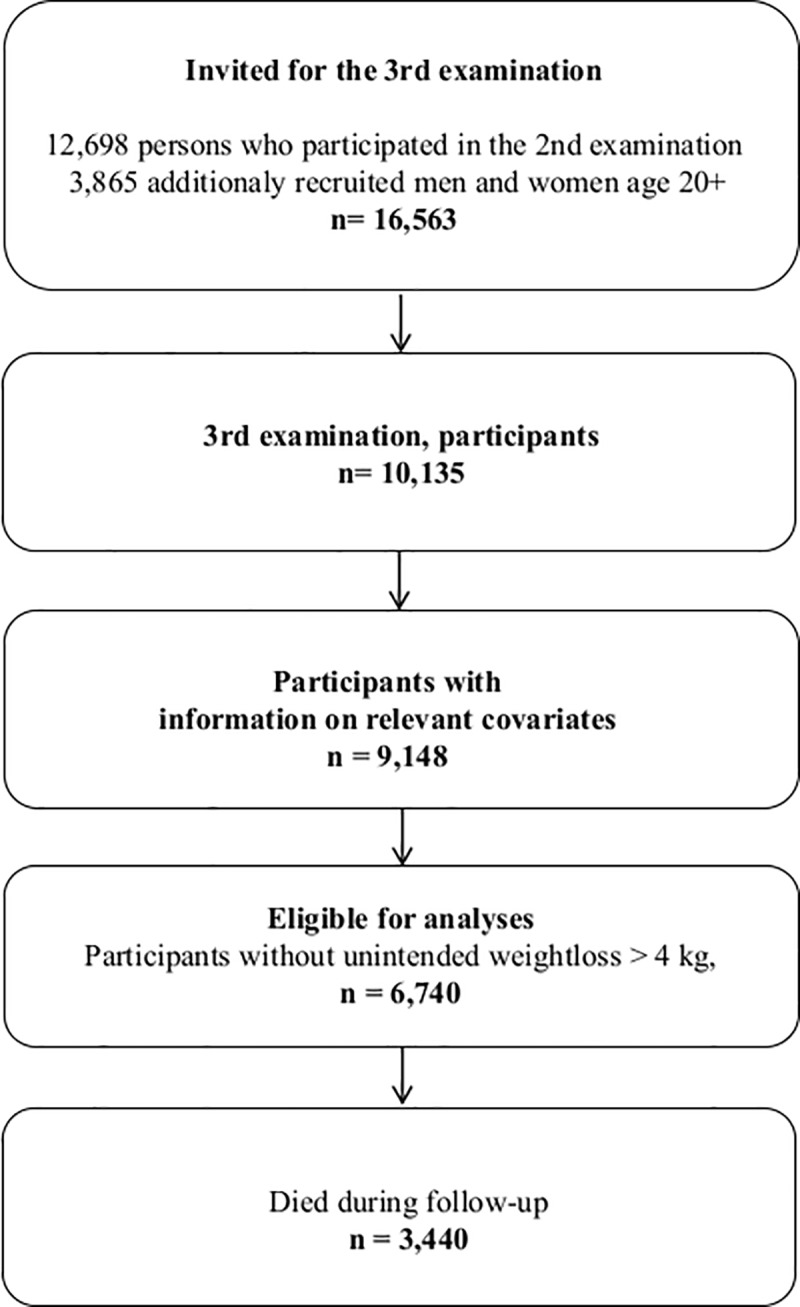
Flow chart depicting the construction of the eventually eligible study sample out of the original cohort of the Copenhagen City Heart Study.

The present analyses uses the data obtained at the 3^rd^ examination, carried out in 1991–94 [25], to which 16,563 were invited and 10,135 (61%) participated. This examination was defined as the baseline examination in the present study. Information on the relevant variables were available for 9,148 participants (Fig 1). This examination included additional questions addressing general well-being (score based on 17 items), and questions about various aspects of BWC not addressed in preceding or subsequent cycles (with abbreviations used in the following in parenthesis):

- ‘Do you care for your body weight in daily life?’ (‘Care?’)
- ‘Do you think it will be good for your health to lose weight?’ (‘Good?’)
- ‘Are you currently trying to slim?’(‘Trying?’)
- ‘Have you tried to slim during the past 15 years?’ (‘Tried?’)

The participants were also asked ‘What did you weigh half-a-year ago?’ and ‘Have you experienced a weight loss of more than 4 kg during a year while not trying to slim?’ The former question defined the pre-baseline BMI and the recent weight changes used in the analyses by subtracting the reported recalled weight from the measured weight at the 3^rd^ examination. In order to limit the possibility that underlying diseases or other reasons for unintentional weight loss, likely associated with mortality, confounded the associations, the analyses were restricted to those who responded ‘No’ to the question about unintentional weight loss, which reduced the study sample to 6,740 for whom required information was available (Fig 1).

The participants were followed-up until November 14, 2014 via the Central Person Register, which includes continuous updates on vital status of all citizens in Denmark. Since previous studies did not indicate that specific causes of death were responsible for the excess mortality after weight loss, we did not request data from the Cause of Death Register.

The original collection of data during the examinations of the Copenhagen City Heart Study required informed consent. Since the present study involved only re-analyses of pre-existing anonymous data from these examinations, no permissions were required according to Danish law from the ethical committees or the data protection agency.

The all-cause mortality from the baseline time of the 3^rd^ examination was analysed by Cox regression models, stratified by sexand using age as the underlying time scale, hence allowing for delayed entry to the estimation. The estimated model produced hazard ratios (HRs) with 95% confidence intervals and allowed null hypothesis testing of main as well as interaction effects. The key null hypotheses state that the ‘Yes’ or ‘No’ responses to the BWC questions are unrelated to all-cause mortality in normal and overweight people irrespective of whether they have lost, maintained or gained in body weight during the past 6 months.

Thus, separate analyses were conducted in normal weight groups (BMI < 25.0 kg/m^2^, here defined as ‘normal’ despite the inclusion in this range of the ‘underweight’ people, usually defined as BMI < 18.5 kg/m^2^) and overweight (BMI ≥ 25.0 kg/m^2^). The division was based on the pre-baseline BMI before the body weight change, i.e. based on the participants’ response to the question about how much they weighed half-a-year earlier. All the models were run without (‘crude’) and with (‘adjusted’) the co-variables, which included pre-baseline BMI (as continuous variable, but truncated according to the two groups allowing them to be used as linear variables), educational level, life style factors and the well-being score. Separate models estimated the associations of mortality with the responses, ‘Yes’ or ‘No’, to each of the four BWC questions in combination with the body weight changes the past half year. Two types of models estimated these associations; one in which the body weight changes were entered as continuous variables and one in which the body weight changes were divided into three categories, weight loss (‘Loss’; BMI change < 0.0 kg/m^2^), stable or small weight gain (‘Stable’; BMI change 0.0–0.8 kg/m^2^), and greater weight gain (‘Gain’; BMI change > 0.8 kg/m^2^) with approximate equal number of deaths in these categories. In the models with the body weight change as a continuous variable, the association was estimated by a smooth restricted cubic spline function with a reference point set at the HR at exactly no weight change in the groups answering ‘No’ to the BWC questions. These models allowed testing for linearity and similarity of the shape of the associations for the ‘Yes’ and ‘No’ groups. In the models with body weight changes as categories, the combination of the answer ‘No’ to the BWC questions and ‘Stable’ weight the past half year was expected to have the lowest mortality and was chosen as the reference in the estimation of associations of the five other combinations with mortality. These models were tested for interactions between the responses to the BWC questions and the body weight change categories in associations with mortality.

## Results

During the follow-up period of about 22 years, a total of 3,440 out of the 6,740 (51%) participants died. The mean pre-baseline BMI was 25.4 kg/m^2^ with a SD of 4.2 kg/m^2^, the mean BMI change prior to baseline was 0.4 kg/m^2^ with a SD of 1.4 kg/m^2^, with weight loss in 2,049 (30.4%), stable weight in 2,324 (34.5%) and weight gain in 2,367 (35.1%). A ‘Yes’ response to the BWC questions was provided by 3,364 (49.9%) about ‘Care?’, by 2,580 (38.4%) about ‘Good?’, by 871 (13.0%) about ‘Trying?’, and by 1,949 (29.0%) about ‘Tried?’ Number of participants, number of deaths and mean (SD) of the BMI-measures (pre-baseline and change) were distributed across categories of the variables as shown in descriptive supplementary tables (for single categories in S1 and S2 Tables, and across combinations of categories of normal weight and overweight groups, weight changes prior to baseline, BWC questions and the responses to these questions in S3A–S3D Table). Each combination has a sizeable number of participants and deaths with a few exceptions, making the estimates for these groups rather uncertain (number of deaths less than 20 in few cells in S3B and S3C Table).

Table 1 shows the crude and adjusted HRs estimated by the Cox regression analyses of the pre-baseline BMI, the categories of changes in BMI and the responses to the four BWC questions separately for the normal weight and the overweight groups. The HRs were only minimally altered by the adjustment except for the HRs of the responses to the BWC questions in the normal weight group, where all HRs increased and changed statistical significance as judged from the confidence intervals (‘Care’ lost significance and ‘Good’ and ‘Trying’ became significant). As expected, the association of pre-baseline BMI with mortality was below HR = 1 in normal weight group and above in the overweight group. Weight loss was followed by significantly greater mortality in the normal weight group, but not in the overweight group, and weight gain was not associated with significantly altered mortality in either of these groups. In the normal weight group, the adjusted HRs were significantly increased for those responding ‘Yes’ compared to ‘No’ to the BWC questions, ‘Good?’ and ‘Trying?’, but not statistically different for the BWC questions, ‘Care’ and ‘Tried’. In the overweight group, none of the responses to the BWC questions were significantly associated with different HRs in the adjusted analyses.

**Table 1:** All-cause mortality according to pre-baseline BMI, body weight changes and body weight control questions.

Pre-baseline BMI group^a^	Characteristic	Crude HR (95% CI)	Adjusted^b^ HR (95% CI)
**< 25 kg/m**^**2**^	**Pre-baseline BMI**	0.95 (0.93, 0.98)	0.97 (0.94, 1.00)
	**Weight change group**^**c**^		
	Loss	1.29 (1.15, 1.46)	1.24 (1.10, 1.41)
	Stable	1.00	1.00
	Gain	0.94 (0.83, 1.07)	0.96 (0.85, 1.09)
	**Body weight control questions**^**d**^		
	**‘Care?** Yes	0.79 (0.71, 0.87)	0.97 (0.86, 1.08)
	No	1.00	1.00
	**‘Good?’** Yes	1.02 (0.86, 1.21)	1.24 (1.03, 1.49)
	No	1.00	1.00
	**‘Trying?’** Yes	1.10 (0.84, 1.43)	1.34 (1.02, 1.77)
	No	1.00	1.00
	**‘Tried?** Yes	0.96 (0.80, 1.15)	1.14 (0.95, 1.37)
	No		
**≥ 25 kg/m**^**2**^	**Pre-baseline BMI**	1.03 (1.01, 1.04)	1.03 (1.01, 1.04)
	**Weight change group**^**c**^		
	Loss	1.11 (1.00, 1.25)	1.04 (0.93, 1.17)
	Stable	1.00	1.00
	Gain	1.03 (0.92, 1.15)	1.03 (0.92, 1.15)
	**Body weight control questions**^**d**^		
	**‘Care?’** Yes	0.90 (0.82, 0.99)	0.98 (0.90, 1.08)
	No	1.00	1.00
	**‘Good?’** Yes	1.04 (0.95, 1.14)	1.00 (0.90, 1.11)
	No	1.00	1.00
	**‘Trying?’** Yes	1.08 (0.96, 1.22)	1.08 (0.95, 1.23)
	No	1.00	1.00
	**‘Tried?’** Yes	1.07 (0.97, 1.18)	1.08 (0.97, 1.20)
	No	1.00	1.00

^a^Pre-baseline BMI is self-reported BMI 6 months prior to the examination.

^b^Analyses were adjusted for pre-baseline BMI, weight change group, educational level, smoking status, alcohol consumption, leisure time physical activity and well-being; stratified by sex; age as time-axis.

^c^Loss’ is < 0 kg/m^2^ change during the recent 6 months, ‘Stable’ is 0–0.8 kg/m^2^ change, ‘Gain’ is > 0.8 kg/m^2^ change.

^d^The questions are ‘Do you care for your body weight in daily life?’ (‘Care?’), ‘Do you think it will be good for your health to lose weight?’ (‘Good?’), ‘Are you currently trying to slim?’(‘Trying?’), ‘Have you tried to slim during the past 15 years?’ (‘Tried?’).

As seen in the S4 Table, the men had a significantly higher mortality than the women, which was accounted for in the Cox regression models using sex for internal stratification. All other co-variables, used for the adjustment of the HRs, were significantly associated with mortality in the expected direction, both when analysed separately and together (greater mortality with lower than higher education, with rare than moderate frequency of alcohol drinking, with daily than with monthly and weekly drinking, with more than less smoking, with less than more physical activity, and with poorer than better well-being).

The following results address, separately for the normal weight and the overweight group, the association of mortality, assessed by HRs, with the combination of the responses to the four BWC questions and the weight changes prior to baseline, either as continuous or categorical variables.

**Fig 2:**
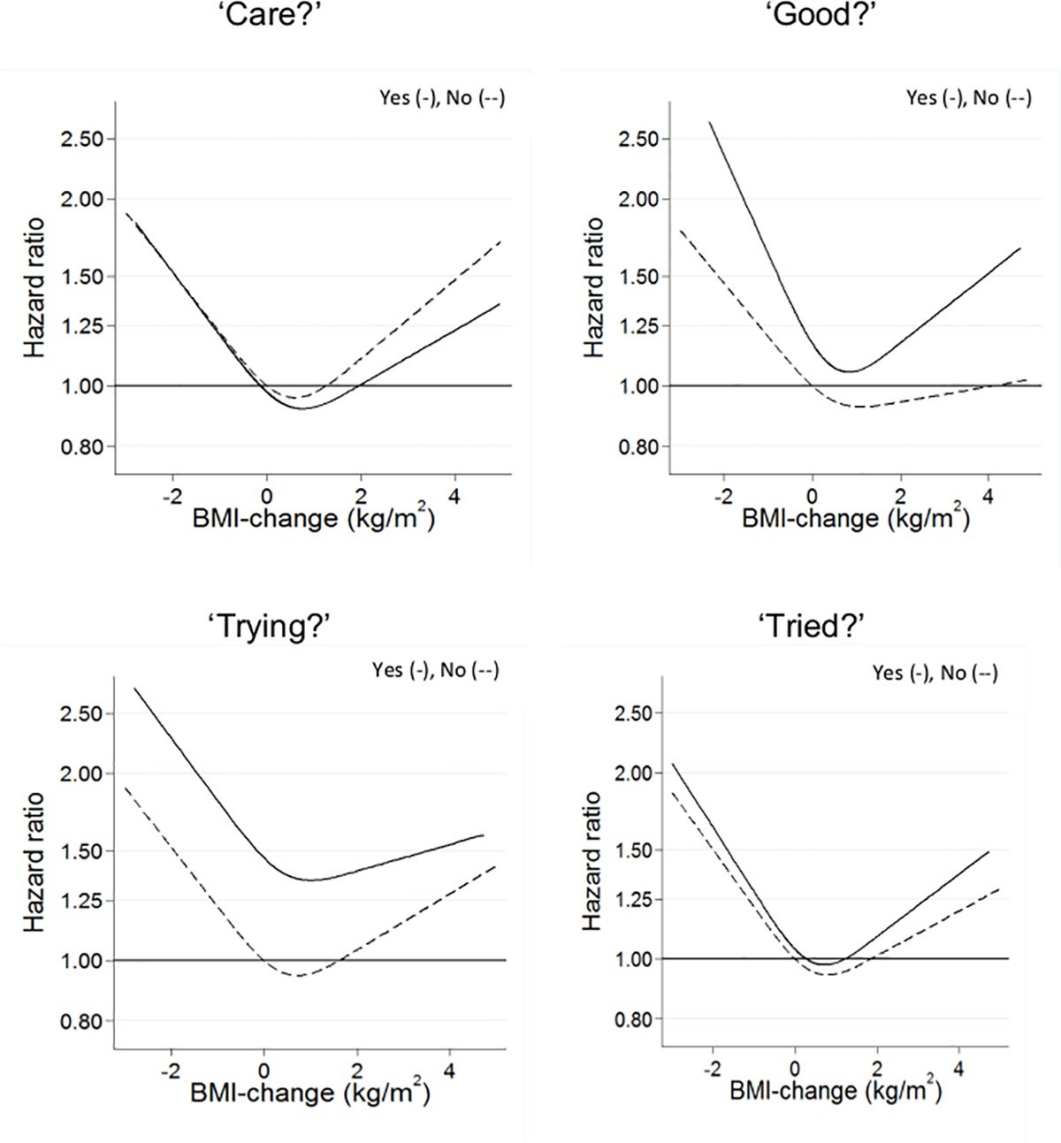
Body weight control, changes in body weight and all-cause mortality in relation to body weight control questions in the normal weight group; Yes (-), No (--).

In the normal weight group, the spline analyses of the weight change as continuous variable showed a V-shaped non-linear association between weight changes for both responses to all four BWC questions (Fig 2). The departure from linearity was statistically significant for all associations (P values ranging between 0.0000014 and 0.032), except for the small group answering ‘Yes’ to the BWC question ‘Trying?’ (P value 0.39). There were no statistically significant differences between the shapes of the spline associations for the groups answering ‘Yes’ and ‘No’ to each of the four BWC questions (all P values between 0.38 and 0.91), indicating no significant interaction between them and the prior weight changes.

**Fig 3:**
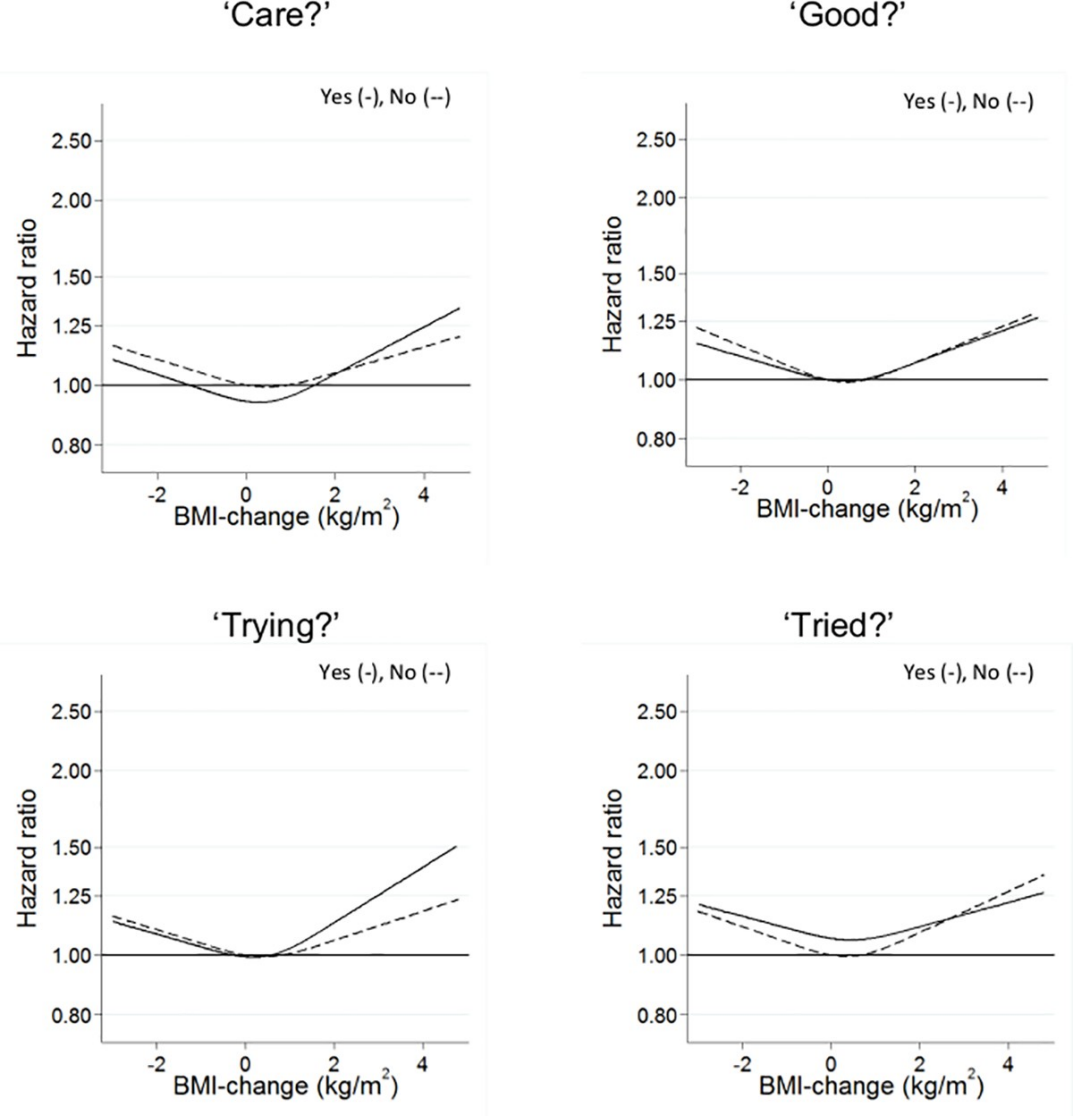
Body weight control, changes in body weight and all-cause mortality in relation to body weight control questions in an overweight population; Yes (-), No (--).

The results of the corresponding analyses for the overweight group are shown in Fig 3. The spline analyses of these groups also showed non-linear, V-shaped associations between weight change and mortality, but overall much less pronounced than in the normal weight groups, and with only half of the departures from linearity being significant (all P values ranging between 0.012 and 0.22). There were no statistically significant differences between the shapes of the spline associations for the groups answering ‘Yes’ and ‘No’ to each of the four BWC questions (all P values between 0.74 and 0.90).

The results of the analyses using the prior weight changes as categories, shown in Table 2, confirmed the overall pattern of results shown by the spline modelling.

**Table 2:** All-cause mortality according to body weight changes combined with body weight control questions.

				Weight change group^a^			
Pre-baseline	Body weight control	Answer to the	‘Loss’^d^	‘Stable’^d^	‘Gain’^d^	*p-value*^e^
BMI group^b^	question^c^	question	HR (95% CI)	HR (95% CI)	HR (95% CI)	
**< 25 kg/m**^**2**^	**‘Care?’**	Yes	1.20 (0.99, 1.45)	1.12 (0.95, 1.33)	0.97 (0.81, 1.15)	
		No	1.40 (1.19, 1.64)	1.00	1.09 (0.90, 1.31)	0.07
	**‘Good?’**	Yes	1.36 (0.79, 2.33)	0.95 (0.68, 1.33)	1.32 (1.05, 1.66)	
		No	1.24 (1.09, 1.40)	1.00	0.90 (0.78, 1.03)	0.09
	**‘Trying?’**	Yes	1.17 (0.55, 2.47)	1.81 (1.09, 2.99)	1.25 (0.87, 1.78)	
		No	1.26 (1.11, 1.42)	1.00	0.95 (0.84, 1.09)	0.32
	**‘Tried?’**	Yes	1.39 (0.93, 2.07)	1.19 (0.86, 1.63)	1.07 (0.83, 1.38)	
		No	1.25 (1.10, 1.42)	1.00	0.95 (0.83, 1.09)	0.95
**≥ 25 kg/m**^**2**^	**‘Care?’**	Yes	1.01 (0.86, 1.19)	0.96 (0.81, 1.13)	1.01 (0.86, 1.18)	
		No	1.03 (0.88, 1.21)	1.00	1.00 (0.85, 1.17)	0.88
	**‘Good?’**	Yes	1.01 (0.85, 1.19)	0.88 (0.74, 1.04)	0.98 (0.84, 1.14)	
		No	0.95 (0.80, 1.12)	1.00	0.92 (0.77, 1.10)	0.19
	**‘Trying?’**	Yes	1.11 (0.91, 1.36)	0.92 (0.69, 1.22)	1.15 (0.94, 1.40)	
		No	1.02 (0.90, 1.15)	1.00	0.99 (0.88, 1.12)	0.43
	**‘Tried?’**	Yes	1.12 (0.94, 1.33)	0.96 (0.79, 1.18)	1.10 (0.94, 1.29)	
		No	1.00 (0.88, 1.15)	1.00	0.97 (0.85, 1.11)	0.39

^a^Loss’ is < 0 kg/m^2^ change during the recent 6 months, ‘Stable’ is 0–0.8 kg/m^2^ change, ‘Gain’ is > 0.8 kg/m^2^ change.

^b^Pre-baseline BMI is self-reported BMI 6 months prior to the examination.

^c^The questions are ‘Do you care for your body weight in daily life?’ (‘Care?’), ‘Do you think it will be good for your health to lose weight?’ (‘Good?’), ‘Are you currently trying to slim?’(‘Trying?’), ‘Have you tried to slim during the past 15 years?’ (‘Tried?’).

^d^Analyses were adjusted for pre-baseline BMI, weight change group, educational level, smoking status, alcohol consumption, leisure time physical activity and well-being; stratified by sex; age as time-axis.

^e^The displayed p-values are from corresponding interaction analyses.

In the normal weight group, weight loss was associated with greater mortality compared with the reference group (‘Stable’ weight and ‘No’ to the BWC questions), which according to the confidence intervals was significant for all the groups answering ‘No’ to the BWC questions. Weight gain showed weaker and less consistent association with mortality, with only one being significant (‘Yes’ to BWC question ‘Good?’). The small group answering ‘Yes’ to the BWC question ‘Trying?’ in the ‘Stable’ group showed a significantly greater HR than those answering ‘No’.

In the overweight group, the categorical analyses were also in agreement with the spline analyses, and showed only weak non-significant differences between the weight change groups, and, in particular, no significant differences related to the BWC questions.

Test for interactions between the responses to the BWC questions and the weight change categories were all not significant (all p-values between 0.07 and 0.95), which corresponds well to the finding of no significant differences by responses to the BWC questions in the shapes of the spline associations shown in Figs 2 and 3.

## Discussion

In this study of individuals from the general population, who reported that they had not suffered from health problems that in the past have induced unintentional weight loss of at least 4 kg during a year, the normal weight people who lost weight experienced a 24% excess mortality compared to those keeping their weight stable, whereas those gaining weight during the past half year did not show excess mortality. The normal weight people who claimed that it would be good for their health to lose weight and those who were currently trying to lose weight also experienced an increased mortality compared to those who did not provide such claims. Besides these findings, the responses to the BWC questions were not associated with mortality. When the responses to the BWC questions were combined with prior weight changes in interaction analyses, we found no consistent and significant modification of the associations with the long-term all-cause mortality. This result was obtained in Cox regression models for normal weight or overweight groups, where sex was used for stratification and age was used as the time axis. The models adjusted the associations for a panel of possibly confounding variables such as pre-baseline BMI, educational level, smoking, alcohol drinking, physical activity and a score of well-being, all of which showed associations with long-term mortality as expected.

With statistical so-called ‘null result’, it is of course necessary to consider whether the statistical power is too low implying a risk of overlooking possibly important associations and interactions. The power of the study was great enough to show the expected associations with the other co-variates, but the width of the confidence intervals must be considered. Thus, we cannot preclude that influences producing true hazard ratios different from the estimated hazard ratios are sources of the actual results within the ranges defined by the confidence limits. However, we find the ranges of the confidence limits depicted in Tables 1 and 2 to be small enough to suggest that there may be no such deviant influences that would be important to consider in public health settings.

Another necessary consideration is whether there is unknown or residual confounding in the analyses or whether there are selection or information biases in play that have reduced the estimated associations and thereby suppressed underlying true and possibly important associations. As always in reanalyses of this sort of long-term observational, longitudinal population-based cohort studies, addressing scientific questions not planned when the data was collected imply unavoidable limitations. They include for example the sparse and uncertain information about the weight changes and the short period for weight changes prior to baseline. Combined information about body composition and physical fitness at baseline would also have been helpful [26]. However, an important argument in favour of the validity of the results is that the data did show the expected mortality patterns regarding all the other co-variables than those probing the attitudes to and experiences with BWC. While admitting that there could still be confounding and biases in addressing the key hypotheses, it should also be acknowledged that it is only by accessing such historical data that we may get any knowledge about the long-term effects.

In the present context, the most important limitation may be that the BWC questions were just asked on the single occasion of the 3^rd^ cycle of the cohort study and that the questions allowed only simple dichotomous answers pertaining to the currently perceived state at that time. Thus, there were no opportunities to investigate what the people meant by their responses and to which extent the expressed attitudes and experiences were persistent, neither before nor the many years after the single examination. The finding that normal weight people claiming that weight loss would be good to their health or that they were currently trying to lose weight experienced a significantly increased mortality irrespective of the thorough adjustments for a broad panel of pertinent co-variables, including pre-baseline BMI and weight changes, is intriguing, but should be replicated and more thoroughly investigated before being translated to a suggested causal relationship.

It should be noted that by adjusting for health behaviours such as smoking, alcohol drinking and physical activity and for well-being, we may have removed some of the effects possibly influenced by the stress of attempting body weight control. We found it more important to control for the possible confounding by these variables than trying to assess their possible mediating role in the investigated association between the responses to the BWC questions and mortality. Thus, it remains possible that a sustained active and successful approach to BWC may reduce the long-term mortality as demonstrated with planned treatments for the patients with obesity [5, 6]. Thus, paying attention to BWC may compensate for adverse effects of weight loss by promoting a healthier behaviour, for example by increased physical activity, which seems to mitigate the excess mortality associated with weight loss in the general population [27], possibly by improving body composition and especially by enhancing cardiorespiratory fitness [26].

Weight loss in the normal weight groups–but not in the overweight groups–was consistently associated with significantly greater mortality, whereas weight gain prior to baseline was not significantly associated with mortality (Table 1). In the combined analyses with stable body weight with answers ‘No’ to the BWC questions as reference (Table 2), the finding in the normal weight group of an increased mortality by weight loss was statistically significant in all the ‘No’ answer groups, but otherwise, there were no consistent pattern in the point estimates of the HRs for the ‘Yes’ versus ‘No’ answers. Correspondingly, there were no significant interactions between weight changes and the responses to the four BWC question, neither when assessed by the shapes of the continuous weight change-based spline functions (Figs 2 and 3) or by the categories of weight changes (Table 2).

Although we believe that we have minimized the risk of underlying confounding by unknown diseases and unhealthy behaviours by excluding all who reported a preceding even moderate unintentional weight loss (> 4 kg during one year) and by controlling for educational level, health behaviours and the well-being score, we admit that such confounding remains a possibility. The absence of a consistently increased mortality by weight gain through all groups may be due to the rather limited gain observed during the half year preceding the examination. Whereas there were no indications in the present results that the answers to the BWC questions modified the association between weight changes and mortality, it is possible that this may be true for more substantial weight changes.

The present study focussed on the crude outcome, long-term all-cause mortality. The absence of its associations with the responses to the BWC questions in most cases in no way excludes that there may be several other health effects of the attitudes and experiences behind these responses, including both body weight control as well as behaviours related or unrelated to body weight control [26]. With the current results at hand, questions about cause-specific mortality and effects on other health indicators may be relevant, which is for later studies to elucidate.

Given the conditions of the study and its results, we conclude that in the general population, the long-term mortality of both normal weight and overweight individuals do not depend on their attitudes and experiences with BWC, however with the following specific exception; normal weight people claiming that it would be good for their health to lose weight or who were actually trying to lose weight showed increased mortality compared with those who did not provided such claims. These relations were not dependent on whether the people had kept their body weight stable, lost or gained body weight during the prior months. Overall, the findings did not contribute to explain the observed associations with body weight changes with mortality in the general population.

## Supporting information

S1 Table Characteristics of the eligible sample according to body weight changes and body weight control questions. (DOCX)

S2 Table Characteristics of the eligible sample according to baseline co-variables. (DOCX)

S3 Table Characteristics of the eligible sample according to body weight changes combined with body weight control question ‘Do you care for your body weight?’. (DOCX)

S4 Table All-cause mortality according to baseline co-variables. (DOCX)
