# Correction: Attitudes to and experiences with body weight control and changes in body weight in relation to all-cause mortality in the general population

**DOI:** 10.1371/journal.pone.0222590

**Published:** 2019-09-11

**Authors:** The PLOS ONE Staff

There are errors in the Author Contributions. The correct contributions are:

**Conceptualization:** Thorkild I.A. Sørensen

**Data curation:** Lars Ängquist

**Formal analysis:** Lars Ängquist

**Funding acquisition:** Merete Appleyard, Peter Schnohr, Gorm B. Jensen, Thorkild I.A. Sørensen

**Investigation:** Merete Appleyard, Peter Schnohr, Gorm B. Jensen, Thorkild I.A. Sørensen

**Methodology:** Lars Ängquist, Peter Schnohr, Gorm B. Jensen, Thorkild I.A. Sørensen

**Project administration:** Merete Appleyard, Peter Schnohr, Gorm B. Jensen, Thorkild I.A. Sørensen

**Resources:** Merete Appleyard, Peter Schnohr, Gorm B. Jensen, Thorkild I.A. Sørensen

**Supervision:** Peter Schnohr, Gorm B. Jensen, Thorkild I.A. Sørensen

**Visualization:** Camilla S. Morgen, Lars Ängquist

**Writing–original draft:** Camilla S. Morgen, Thorkild I.A. Sørensen

**Writing–reviewing & editing:** Camilla S. Morgen, Lars Ängquist, Peter Schnohr, Gorm B. Jensen, Thorkild I.A. Sørensen.

The publisher apologizes for the errors.
