# Supplementary material for: Attitudes to and experiences with body weight control and changes in body weight in relation to all-cause mortality in the general population
Source: PLoS One. 2019 Aug 15;14(8):e0220838. doi: 10.1371/journal.pone.0220838 (PMC6695162; doi:10.1371/journal.pone.0220838)
Supplement: S1 Table — (DOCX) [file pone.0220838.s001.docx]

**S1 Table. Characteristics of the eligible sample according to body weight changes and body weight control questions^a^**

|  | | **Participants** | |  | **Deaths** | |  | **Pre-baseline BMI** |  |  | | **BMI change** | |  |
| --- | --- | --- | --- | --- | --- | --- | --- | --- | --- | --- | --- | --- | --- | --- |
|  | | n | % |  | n | ^%^ |  | Mean (SD)^c^ |  | | Mean (SD) | |  |  |
|  |  | |  |  |  |  |  |  |  | |  | |  |  |
| **Weight change group^d^** |  | |  |  |  |  |  |  |  | |  | |  |  |
| Loss | 2,049 | | 30.4 |  | 1,143 | 33.2 |  | 26.1 (4.7) |  | | -0.9 (1.2) | |  |  |
| Stable | 2,324 | | 34.5 |  | 1,150 | 33,4 |  | 24.7 (3.9) |  | | 0.4 (0.2) | |  |  |
| Gain | 2,367 | | 35.1 |  | 1,147 | 33.3 |  | 25.5 (4.1) |  | | 1.7 (1.0) | |  |  |
| Total | 6,740 | | 100.0 |  | 3,440 | 100.0 |  |  |  | |  | |  |  |
| **Body weight control question^b^** |  | |  |  |  |  |  |  |  | |  | |  |  |
| **‘Care?’** |  | |  |  |  |  |  |  |  | |  | |  |  |
| No | 3,374 | | 50.1 |  | 1,708 | 49,7 |  | 25.2 (4.4) |  | | 0.4 (1.3) | |  |  |
| Yes | 3,364 | | 49.9 |  | 1,731 | 50.3 |  | 25.7 (4.1) |  | | 0.5 (1.4) | |  |  |
| Total | 6,738 | | 100.0 |  | 3,439 | 100.0 |  |  |  | |  | |  |  |
|  |  | |  |  |  |  |  |  |  | |  | |  |  |
| **‘Good?’** |  | |  |  |  |  |  |  |  | |  | |  |  |
| No | 4,142 | | 61.6 |  | 2,147 | 62.6 |  | 23.5 (3.0) |  | | 0.3 (1.1) | |  |  |
| Yes | 2,580 | | 38.4 |  | 1,284 | 37.4 |  | 28.5 (4.1) |  | | 0.6 (1.6) | |  |  |
| Total | 6,722 | | 100.0 |  | 3,431 | 100.0 |  |  |  | |  | |  |  |
|  |  | |  |  |  |  |  |  |  | |  | |  |  |
| **‘Trying?’** |  | |  |  |  |  |  |  |  | |  | |  |  |
| No | 5,846 | | 87.0 |  | 3,054 | 89.2 |  | 25.0 (4.0) |  | | 0.5 (1.3) | |  |  |
| Yes | 871 | | 13.0 |  | 370 | 10.8 |  | 28.4 (4.9) |  | | 0.3 (1.9) | |  |  |
| Total | 6,717 | | 100.0 |  | 3,424 | 100.0 |  |  |  | |  | |  |  |
|  |  | |  |  |  |  |  |  |  | |  | |  |  |
| **‘Tried?’** |  | |  |  |  |  |  |  |  | |  | |  |  |
| No | 4,763 | | 71.0 |  | 2,641 | 77.1 |  | 24.5 (3.7) |  | | 0.4 (1.2) | |  |  |
| Yes | 1,949 | | 29.0 |  | 784 | 22.9 |  | 27.6 (4.7) |  | | 0.6 (1.7) | |  |  |
| Total | 6,712 | | 100.0 |  | 3,425 | 100.0 |  |  |  | |  | |  |  |
| ^a^The numbers do not sum up to 6,740 in each question, since the sample was not restricted to have answered all body weight related questions, but answering at least one of the questions was required.  ^b^The questions are ‘Do you care for your body weight in daily life?’ (‘Care?’), ‘Do you think it will be good for your health to lose weight?’ (‘Good?’), ‘Are you currently trying to slim?’(‘Trying?’), ‘Have you tried to slim during the past 15 years?’ (‘Tried?’).  ^c^Pre-baseline BMI is self-reported BMI 6 months prior to the examination.  ^d’^Loss’ is < 0 kg/m^2^ change during the recent 6 months, ‘Stable’ is 0-0.8 kg/m^2^ change, ‘Gain’ is > 0.8 kg/m^2^ change. | | | | | | | | | | | | | | |
