# Supplementary material for: Attitudes to and experiences with body weight control and changes in body weight in relation to all-cause mortality in the general population
Source: PLoS One. 2019 Aug 15;14(8):e0220838. doi: 10.1371/journal.pone.0220838 (PMC6695162; doi:10.1371/journal.pone.0220838)
Supplement: S2 Table — (DOCX) [file pone.0220838.s002.docx]

**S2 Table. Characteristics of the eligible sample according to baseline co-variables**

|  | **Participants** | | |  | **Deaths** | |  | | **Pre-baseline BMI^a^** |  | | | **BMI change** |  |  |
| --- | --- | --- | --- | --- | --- | --- | --- | --- | --- | --- | --- | --- | --- | --- | --- |
| **Characteristics** | n | % | |  | n | % | |  | Mean (SD) | |  | Mean (SD) | | |  |
| **Total** | 6,740 | | 100 |  | 3,440 | 100 | |  | 25.4 (4.2) | |  | 0.4 (1.4) | | |  |
| **Sex** |  | |  |  |  |  | |  |  | |  |  | | |  |
| Women | 3,549 | | 52.7 |  | 1,703 | 49.5 | |  | 25.1 (4.6) | |  | 0.5 (1.5) | | |  |
| Men | 3,191 | | 47.3 |  | 1,737 | 50.5 | |  | 25.9 (3.8) | |  | 0.3 (1.2) | | |  |
| **Age** |  | |  |  |  |  | |  |  | |  |  | | |  |
| 20 -< 35 | 602 | | 8.9 |  | 18 | 0.5 | |  | 22.7 (3.2) | |  | 0.5 (1.2) | | |  |
| 35 -< 55 | 1,859 | | 27.6 |  | 313 | 9.1 | |  | 24.9 (4.3) | |  | 0.5 (1.4) | | |  |
| 55 -< 75 | 3,434 | | 51.0 |  | 2,301 | 66.9 | |  | 26.1 (4.3) | |  | 0.4 (1.4) | | |  |
| ≥ 75 | 845 | | 12.5 |  | 808 | 23.5 | |  | 25.7 (3.8) | |  | 0.4 (1.3) | | |  |
| **Educational level (years)** |  | |  |  |  |  | |  |  | |  |  | | |  |
| 6 – 8 | 2,356 | | 35.0 |  | 1,634 | 47.5 | |  | 26.5 (4.5) | |  | 0.4 (1.5) | | |  |
| 9 – 10 | 2,663 | | 39.5 |  | 1,358 | 39.5 | |  | 25.5 (4.2) | |  | 0.4 (1.4) | | |  |
| ≥ 11 | 1,721 | | 25.5 |  | 448 | 13.0 | |  | 23.8 (3.4) | |  | 0.5 (1.1) | | |  |
| **Smoking status** |  | |  |  |  |  | |  |  | |  |  | | |  |
| Never smoker | 1,759 | | 26.1 |  | 693 | 20.2 | |  | 25.6 (4.5) | |  | 0.5 (1.3) | | |  |
| Former smoker | 1,819 | | 27.0 |  | 973 | 28.3 | |  | 26.0 (4.1) | |  | 0.5 (1.4) | | |  |
| Light smoker (1-14 cigarettes/day) | 1,311 | | 19.5 |  | 711 | 20.7 | |  | 24.5 (3.9) | |  | 0.4 (1.2) | | |  |
| Heavy smoker (≥14 cigarettes/day) | 1,851 | | 27.5 |  | 1,063 | 30.9 | |  | 25.3 (4.3) | |  | 0.3 (1.5) | | |  |
| **Alcohol consumption** |  | |  |  |  |  | |  |  | |  |  | | |  |
| Never/almost never | 1,136 | | 16.9 |  | 681 | 19.8 | |  | 26.3 (4.8) | |  | 0.4 (1.5) | | |  |
| Monthly | 1,765 | | 26.2 |  | 821 | 23.9 | |  | 25.4 (4.5) | |  | 0.5 (1.5) | | |  |
| Weekly | 2,229 | | 33.1 |  | 889 | 25.8 | |  | 25.0 (4.0) | |  | 0.5 (1.3) | | |  |
| Daily | 1,610 | | 23.9 |  | 1,049 | 30.5 | |  | 25.4 (3.8) | |  | 0.3 (1.2) | | |  |
| **Leisure time physical activity** |  | |  |  |  |  | |  |  | |  |  | | |  |
| < 2 hours per week | 800 | | 11.9 |  | 503 | 14.6 | |  | 26.7 (5.1) | |  | 0.4 (1.6) | | |  |
| 2 - 4 hours per week, light | 3,633 | | 53.9 |  | 1,895 | 55.1 | |  | 25.6 (4.3) | |  | 0.5 (1.3) | | |  |
| 2 - 4 hours per week, moderate | 2,079 | | 30.9 |  | 965 | 28.1 | |  | 24.8 (3.8) | |  | 0.5 (1.3) | | |  |
| > 4 hours per week | 228 | | 3.4 |  | 77 | 2.2 | |  | 24.8 (3.8) | |  | 0.3 (1.2) | | |  |
| **Well-being** |  | |  |  |  |  | |  |  | |  |  | | |  |
| Good | 2,858 | | 42.4 |  | 1,470 | 42.7 | |  | 25.3 (3.9) | |  | 0.4 (1.2) | | |  |
| Moderate | 1,689 | | 25.1 |  | 809 | 23.5 | |  | 25.3 (4.1) | |  | 0.4 (1.4) | | |  |
| Fair | 1,414 | | 21.0 |  | 692 | 20.1 | |  | 25.5 (4.6) | |  | 0.5 (1.7) | | |  |
| Poor | 779 | | 11.6 |  | 469 | 13.6 | |  | 26.0 (4.9) | |  | 0.4 (1.4) | | |  |
| ^a^Pre-baseline BMI is self-reported BMI 6 months prior to the examination.  ^b’^Loss’ is < 0 kg/m^2^ change during the recent 6 months, ‘Stable’ is 0-0.8 kg/m^2^ change, ‘Gain’ is > 0.8 kg/m^2^ change. | | | | | | | | | | | | | | | |
