# Supplementary material for: Attitudes to and experiences with body weight control and changes in body weight in relation to all-cause mortality in the general population
Source: PLoS One. 2019 Aug 15;14(8):e0220838. doi: 10.1371/journal.pone.0220838 (PMC6695162; doi:10.1371/journal.pone.0220838)
Supplement: S3 Table — (DOCX) [file pone.0220838.s003.docx]

**S3 Table A. Characteristics of the eligible sample according to body weight changes combined with body weight control question ‘Do you care for your body weight?’**

| **Pre-baseline** |  | **Weight change group ^b^** |  | **Answer to the body** |  | **Participants** | |  | **Deaths** | |  | **Pre-baseline BMI** |  | **BMI change** |
| --- | --- | --- | --- | --- | --- | --- | --- | --- | --- | --- | --- | --- | --- | --- |
| **BMI group^a^** |  |  |  | **weight control question** |  | n | % |  | n | % |  | Mean (SD) |  | Mean (SD) |
| **< 25 kg/m^2^** |  | Loss |  | No |  | 583 | 60.9 |  | 305 | 63.2 |  | 22.2 (2.0) |  | -0.7 (0.7) |
|  |  |  |  | Yes |  | 374 | 39.1 |  | 178 | 36.9 |  | 22.8 (1.7) |  | -0.6 (0.6) |
|  |  |  |  | Total |  | 957 | 100.0 |  | 483 | 100.0 |  | 22.4 (1.9) |  | -0.6 (0.7) |
|  |  |  |  |  |  |  |  |  |  |  |  |  |  |  |
|  |  | Stable |  | No |  | 729 | 53.9 |  | 291 | 50.4 |  | 21.9 (1.9) |  | 0.4 (0.2) |
|  |  |  |  | Yes |  | 623 | 46.1 |  | 286 | 49.6 |  | 22.4 (1.7) |  | 0.4 (0.2) |
|  |  |  |  | Total |  | 1,352 | 100.0 |  | 577 | 100.0 |  | 22.1 (1.8) |  | 0.4 (0.2) |
|  |  |  |  |  |  |  |  |  |  |  |  |  |  |  |
|  |  | Gain |  | No |  | 513 | 44.0 |  | 191 | 43.6 |  | 22.0 (1.9) |  | 1.5 (0.8) |
|  |  |  |  | Yes |  | 654 | 56.0 |  | 247 | 56.4 |  | 22.6 (1.6) |  | 1.5 (1.0) |
|  |  |  |  | Total |  | 1,167 | 100.0 |  | 438 | 100.0 |  | 22.3 (1.8) |  | 1.5 (0.9) |
|  |  |  |  |  |  |  |  |  |  |  |  |  |  |  |
| **≥ 25 kg/m^2^** |  | Loss |  | No |  | 504 | 46.2 |  | 314 | 47.7 |  | 29.3 (3.9) |  | -1.0 (1.6) |
|  |  |  |  | Yes |  | 586 | 53.8 |  | 345 | 52.4 |  | 29.5 (3.8) |  | -1.2 (1.4) |
|  |  |  |  | Total |  | 1,090 | 100.0 |  | 659 | 100.0 |  | 29.4 (3.8) |  | -1.1 (1.5) |
|  |  |  |  |  |  |  |  |  |  |  |  |  |  |  |
|  |  | Stable |  | No |  | 489 | 50.3 |  | 278 | 48.5 |  | 28.3 (3.1) |  | 0.4 (0.2) |
|  |  |  |  | Yes |  | 483 | 49.7 |  | 295 | 51.5 |  | 28.2 (2.9) |  | 0.4 (0.2) |
|  |  |  |  | Total |  | 972 | 100.0 |  | 573 | 100.0 |  | 28.3 (3.0) |  | 0.4 (0.2) |
|  |  |  |  |  |  |  |  |  |  |  |  |  |  |  |
|  |  | Gain |  | No |  | 556 | 46.3 |  | 329 | 46.4 |  | 29.1 (3.4) |  | 1.8 (1.0) |
|  |  |  |  | Yes |  | 644 | 53.7 |  | 380 | 53.6 |  | 28.3 (3.1) |  | 1.8 (1.0) |
|  |  |  |  | Total |  | 1,200 | 100.0 |  | 709 | 100.0 |  | 28.7 (3.2) |  | 1.8 (1.0) |

^a^Pre-baseline BMI is self-reported BMI 6 months prior to the examination.

^b^Loss’ is < 0 kg/m^2^ change during the recent 6 months, ‘Stable’ is 0-0.8 kg/m^2^ change, ‘Gain’ is > 0.8 kg/m^2^ change.

**S3 Table B. Characteristics of the eligible sample according to body weight changes combined with body weight control question ‘Do you think it will be good for your health to lose weight?’**

| **Pre-baseline** |  | **Weight change group ^b^** |  | **Answer to the body** |  | **Participants** | |  | **Deaths** | |  | **Pre-baseline BMI** |  | **BMI change** |
| --- | --- | --- | --- | --- | --- | --- | --- | --- | --- | --- | --- | --- | --- | --- |
| **BMI group^a^** |  |  |  | **weight control question** |  | n | % |  | n | % |  | Mean (SD) |  | Mean (SD) |
| **< 25 kg/m^2^** |  | Loss |  | No |  | 895 | 93.5 |  | 469 | 97.1 |  | 22.3 (1.9) |  | -0.6 (0.7) |
|  |  |  |  | Yes |  | 62 | 6.5 |  | 14 | 2.9 |  | 23.8 (1.1) |  | -0.6 (0.7) |
|  |  |  |  | Total |  | 957 | 100.0 |  | 483 | 100.0 |  | 22.4 (1.9) |  | -0.6 (0.7) |
|  |  |  |  |  |  |  |  |  |  |  |  |  |  |  |
|  |  | Stable |  | No |  | 1,206 | 89.5 |  | 539 | 93.6 |  | 22.0 (1.8) |  | 0.4 (0.2) |
|  |  |  |  | Yes |  | 142 | 10.5 |  | 37 | 6.4 |  | 23.5 (1.3) |  | 0.4 (0.2) |
|  |  |  |  | Total |  | 1,348 | 100.0 |  | 576 | 100.0 |  | 22.1 (1.8) |  | 0.4 (0.2) |
|  |  |  |  |  |  |  |  |  |  |  |  |  |  |  |
|  |  | Gain |  | No |  | 898 | 77.1 |  | 342 | 78.3 |  | 22.1 (1.8) |  | 1.4 (0.6) |
|  |  |  |  | Yes |  | 267 | 22.9 |  | 95 | 21.7 |  | 23.3 (1.3) |  | 2.0 (1.5) |
|  |  |  |  | Total |  | 1,165 | 100.0 |  | 437 | 100.0 |  | 22.3 (1.8) |  | 1.5 (0.9) |
|  |  |  |  |  |  |  |  |  |  |  |  |  |  |  |
| **≥ 25 kg/m^2^** |  | Loss |  | No |  | 419 | 38.5 |  | 296 | 44.9 |  | 27.4 (2.4) |  | -1.2 (1.7) |
|  |  |  |  | Yes |  | 669 | 61.5 |  | 364 | 55.2 |  | 30.7 (4.1) |  | -1.1 (1.4) |
|  |  |  |  | Total |  | 1,088 | 100.0 |  | 660 | 100.0 |  | 29.4 (3.9) |  | -1.1 (1.5) |
|  |  |  |  |  |  |  |  |  |  |  |  |  |  |  |
|  |  | Stable |  | No |  | 405 | 41.8 |  | 274 | 48.0 |  | 27.1 (2.1) |  | 0.4 (0.2) |
|  |  |  |  | Yes |  | 564 | 58.2 |  | 297 | 52.0 |  | 29.1 (3.3) |  | 0.4 (0.2) |
|  |  |  |  | Total |  | 969 | 100.0 |  | 571 | 100.0 |  | 28.3 (3.0) |  | 0.4 (0.2) |
|  |  |  |  |  |  |  |  |  |  |  |  |  |  |  |
|  |  | Gain |  | No |  | 319 | 26.7 |  | 227 | 32.2 |  | 27.1 (1.9) |  | 1.5 (0.8) |
|  |  |  |  | Yes |  | 876 | 73.3 |  | 477 | 67.8 |  | 29.2 (3.4) |  | 1.9 (1.0) |
|  |  |  |  | Total |  | 1,195 | 100.0 |  | 704 | 100.0 |  | 28.7 (3.3) |  | 1.8 (1.0) |

^a^Pre-baseline BMI is self-reported BMI 6 months prior to the examination.

^b^Loss’ is < 0 kg/m^2^ change during the recent 6 months, ‘Stable’ is 0-0.8 kg/m^2^ change, ‘Gain’ is > 0.8 kg/m^2^ change.

**S3 Table C. Characteristics of the eligible sample according to body weight changes combined with body weight control question ‘Are you currently trying to slim?’**

| **Pre-baseline** |  | **Weight change group ^b^** |  | **Answer to the body** |  | **Participants** | |  | **Deaths** | |  | **Pre-baseline BMI** |  | **BMI change** |
| --- | --- | --- | --- | --- | --- | --- | --- | --- | --- | --- | --- | --- | --- | --- |
| **BMI group^a^** |  |  |  | **weight control question** |  | n | % |  | n | % |  | Mean (SD) |  | Mean (SD) |
| **< 25 kg/m^2^** |  | Loss |  | No |  | 918 | 96.2 |  | 473 | 98.5 |  | 22.4 (1.9) |  | -0.6 (0.7) |
|  |  |  |  | Yes |  | 36 | 3.8 |  | 7 | 1.5 |  | 23.0 (1.6) |  | -1.0 (0.8) |
|  |  |  |  | Total |  | 954 | 100.0 |  | 480 | 100.0 |  | 22.4 (1.9) |  | -0.6 (0.7) |
|  |  |  |  |  |  |  |  |  |  |  |  |  |  |  |
|  |  | Stable |  | No |  | 1,292 | 95.8 |  | 560 | 97.2 |  | 22.1 (1.8) |  | 0.4 (0.2) |
|  |  |  |  | Yes |  | 57 | 4.2 |  | 16 | 2.8 |  | 22.7 (1.7) |  | 0.5 (0.2) |
|  |  |  |  | Total |  | 1,349 | 100.0 |  | 576 | 100.0 |  | 22.1 (1.8) |  | 0.4 (0.2) |
|  |  |  |  |  |  |  |  |  |  |  |  |  |  |  |
|  |  | Gain |  | No |  | 1,041 | 89.5 |  | 401 | 92.2 |  | 22.3 (1.8) |  | 1.5 (0.9) |
|  |  |  |  | Yes |  | 122 | 10.5 |  | 34 | 7.8 |  | 22.8 (1.3) |  | 1.8 (0.9) |
|  |  |  |  | Total |  | 1,163 | 100.0 |  | 435 | 100.0 |  | 22.3 (1.8) |  | 1.5 (0.9) |
|  |  |  |  |  |  |  |  |  |  |  |  |  |  |  |
| **≥ 25 kg/m^2^** |  | Loss |  | No |  | 806 | 74.2 |  | 524 | 79.9 |  | 28.8 (3.4) |  | -1.0 (1.5) |
|  |  |  |  | Yes |  | 281 | 25.8 |  | 132 | 20.1 |  | 31.2 (4.5) |  | -1.7 (1.5) |
|  |  |  |  | Total |  | 1,087 | 100.0 |  | 656 | 100.0 |  | 29.4 (3.9) |  | -1.1 (1.5) |
|  |  |  |  |  |  |  |  |  |  |  |  |  |  |  |
|  |  | Stable |  | No |  | 845 | 87.3 |  | 518 | 90.6 |  | 28.0 (2.8) |  | 0.4 (0.2) |
|  |  |  |  | Yes |  | 123 | 12.7 |  | 54 | 9.4 |  | 29.7 (3.9) |  | 0.4 (0.2) |
|  |  |  |  | Total |  | 968 | 100.0 |  | 572 | 100.0 |  | 28.3 (3.0) |  | 0.4 (0.2) |
|  |  |  |  |  |  |  |  |  |  |  |  |  |  |  |
|  |  | Gain |  | No |  | 944 | 78.9 |  | 578 | 82.0 |  | 28.5 (3.1) |  | 1.7 (1.0) |
|  |  |  |  | Yes |  | 252 | 21.1 |  | 127 | 18.0 |  | 29.2 (3.6) |  | 2.0 (1.1) |
|  |  |  |  | Total |  | 1,196 | 100.0 |  | 705 | 100.0 |  | 28.7 (3.3) |  | 1.8 (1.0) |

^a^Baseline BMI is self-reported BMI 6 months prior to the examination.

^b^Loss’ is < 0 kg/m^2^ change during the recent 6 months, ‘Stable’ is 0-0.8 kg/m^2^ change, ‘Gain’ is > 0.8 kg/m^2^ change.

**S3 Table D. Characteristics of the eligible sample according to body weight changes combined with body weight control question ‘Have you tried to slim during the past 15 years?’**

| **Pre-baseline** |  | **Weight change group ^b^** |  | **Answer to the body** |  | **Participants** | |  | **Deaths** | |  | **Pre-baseline BMI** |  | **BMI change** |
| --- | --- | --- | --- | --- | --- | --- | --- | --- | --- | --- | --- | --- | --- | --- |
| **BMI group^a^** |  |  |  | **weight control question** |  | n | % |  | n | % |  | Mean (SD) |  | Mean (SD) |
| **< 25 kg/m^2^** |  | Loss |  | No |  | 848 | 89.1 |  | 454 | 94,6 |  | 22.3 (1.9) |  | -0.6 (0.7) |
|  |  |  |  | Yes |  | 104 | 10.9 |  | 26 | 5.4 |  | 23.0 (1.6) |  | -0.7 (0.7) |
|  |  |  |  | Total |  | 952 | 100.0 |  | 480 | 100.0 |  | 22.4 (1.9) |  | -0.6 (0.7) |
|  |  |  |  |  |  |  |  |  |  |  |  |  |  |  |
|  |  | Stable |  | No |  | 1,161 | 86.1 |  | 533 | 92.5 |  | 22.1 (1.9) |  | 0.4 (0.2) |
|  |  |  |  | Yes |  | 187 | 12.9 |  | 43 | 7.5 |  | 22.5 (1.7) |  | 0.4 (0.2) |
|  |  |  |  | Total |  | 1,348 | 100.0 |  | 576 | 100.0 |  | 22.1 (1.8) |  | 0.4 (0.2) |
|  |  |  |  |  |  |  |  |  |  |  |  |  |  |  |
|  |  | Gain |  | No |  | 859 | 74.1 |  | 362 | 83.4 |  | 22.2 (1.9) |  | 1.4 (0.7) |
|  |  |  |  | Yes |  | 301 | 25.9 |  | 72 | 16.6 |  | 22.8 (1.5) |  | 1.8 (1.4) |
|  |  |  |  | Total |  | 1,160 | 100.0 |  | 434 | 100.0 |  | 22.3 (1.8) |  | 1.5 (0.9) |
|  |  |  |  |  |  |  |  |  |  |  |  |  |  |  |
| **≥ 25 kg/m^2^** |  | Loss |  | No |  | 642 | 59.0 |  | 442 | 67.2 |  | 28.4 (3.0) |  | -0.9 (1.5) |
|  |  |  |  | Yes |  | 446 | 41.0 |  | 216 | 32.8 |  | 30.8 (4.5) |  | -1.4 (1.4) |
|  |  |  |  | Total |  | 1,088 | 100.0 |  | 658 | 100.0 |  | 29.4 (3.9) |  | -1.1 (1.5) |
|  |  |  |  |  |  |  |  |  |  |  |  |  |  |  |
|  |  | Stable |  | No |  | 654 | 67.6 |  | 433 | 75.7 |  | 27.7 (2.4) |  | 0.4 (0.2) |
|  |  |  |  | Yes |  | 314 | 32.4 |  | 139 | 24.3 |  | 29.5 (3.7) |  | 0.4 (0.2) |
|  |  |  |  | Total |  | 968 | 100.0 |  | 572 | 100.0 |  | 28.3 (3.0) |  | 0.4 (0.2) |
|  |  |  |  |  |  |  |  |  |  |  |  |  |  |  |
|  |  | Gain |  | No |  | 599 | 50.1 |  | 417 | 59.2 |  | 28.2 (2.9) |  | 1.7 (1.0) |
|  |  |  |  | Yes |  | 597 | 49.9 |  | 288 | 40.9 |  | 29.1 (3.5) |  | 1.9 (1.0) |
|  |  |  |  | Total |  | 1,196 | 100.0 |  | 705 | 100.0 |  | 28.7 (3.3) |  | 1.8 (1.0) |

^a^Pre-baseline BMI is self-reported BMI 6 months prior to the examination.

^b^Loss’ is < 0 kg/m^2^ change during the recent 6 months, ‘Stable’ is 0-0.8 kg/m^2^ change, ‘Gain’ is > 0.8 kg/m^2^ change.
