# Supplementary material for: Attitudes to and experiences with body weight control and changes in body weight in relation to all-cause mortality in the general population
Source: PLoS One. 2019 Aug 15;14(8):e0220838. doi: 10.1371/journal.pone.0220838 (PMC6695162; doi:10.1371/journal.pone.0220838)
Supplement: S4 Table — (DOCX) [file pone.0220838.s004.docx]

**S4 Table. All-cause mortality according to baseline co-variables**

| **Co-variables** | **Crude HR (95% CI)** | **Adjusted^a^ HR (95% CI)** |  |  |  |
| --- | --- | --- | --- | --- | --- |
| **Sex** |  |  |  |  |  |
| Women | 1.00 | 1.00 |  |  |  |
| Men | 1.74 (1.63, 1.86) | 1.63 (1.51, 1.76) |  |  |  |
| **Educational level (years)** |  |  |  |  |  |
| 6 - 8 | 1.00 | 1.00 |  |  |  |
| 9 - 10 | 0.89 (0.83, 0.96) | 0.94 (0.88, 1.02) |  |  |  |
| ≥ 11 | 0.72 (0.64, 0.80) | 0.81 (0.72, 0.90) |  |  |  |
| **Smoking status** |  |  |  |  |  |
| Never smoker | 1.00 | 1.00 |  |  |  |
| Former smoker | 1.20 (1.08, 1.32) | 1.20 (1.08, 1.33) |  |  |  |
| Light smoker (1-14 cigarettes/day) | 1.57 (1.41, 1.74) | 1.55 (1.39, 1.73) |  |  |  |
| Heavy smoker (>14 cigarettes/day) | 2.44 (2.21, 2.70) | 2.27 (2.04, 2.52) |  |  |  |
| **Alcohol consumption** |  |  |  |  |  |
| Never/almost never | 1.00 | 1.00 |  |  |  |
| Monthly | 0.83 (0.75, 0.92) | 0.86 (0.77, 0.95) |  |  |  |
| Weekly | 0.80 (0.73, 0.89) | 0.82 (0.74, 0.92) |  |  |  |
| Daily | 1.10 (0.99, 1.21) | 1.07 (0.96, 1.19) |  |  |  |
| **Leisure time physical activity** |  |  |  |  |  |
| < 2 hours per week | 1.00 | 1.00 |  |  |  |
| 2 - 4 hours per week, light | 0.69 (0.63, 0.76) | 0.85 (0.77, 0.94) |  |  |  |
| 2 - 4 hours per week, moderate | 0.61 (0.55, 0.68) | 0.79 (0.70, 0.88) |  |  |  |
| > 4 hours per week | 0.59 (0.46, 0.74) | 0.77 (0.60, 0.98) |  |  |  |
| **Well-being** |  |  |  |  |  |
| Good | 1.00 | 1.00 |  |  |  |
| Moderate | 1.11 (1.02, 1.21) | 1.08 (0.99, 1.18) |  |  |  |
| Fair | 1.24 (1.06, 1.28) | 1.17 (1.02, 1.21) |  |  |  |
| Poor | 1.68 (1.51, 1.87) | 1.40 (1.26, 1.56) |  |  |  |
| ^a^Analyses were adjusted for – when applicable - pre-baseline BMI, weight change group, educational level, smoking status, alcohol consumption, leisure time physical activity and well-being; stratified by sex (apart for the sex-co-variable); age as time-axis. | | | |  |  |
